# Supplementary material for: Synthesis and Performance of Double-Chain Quaternary Ammonium Salt Glucosamide Surfactants
Source: Molecules. 2022 Mar 26;27(7):2149. doi: 10.3390/molecules27072149 (PMC9000696; doi:10.3390/molecules27072149)
Supplement: Supplementary file 1 [file molecules-27-02149-s001.zip › molecules-1577205-supplementary.pdf]

Supporting Information

Synthesis and Performance of Double-chain Quaternary Ammonium Salt Glucosamide Surfactants

Lifei Zhi*, Xiufang Shi, Erzhuang Zhang, Chuangji Gao, Haocheng Gai, Hui Wang, Zhenmin Liu and Tieming Zhang

College of Chemistry and Biological Engineering, Taiyuan University of Science and Technology, Taiyuan, , 030024, China; sxf769593427@163.com (X.S.); s20202111007@stu.tyust.edu.cn (E.Z.); 201921020119@stu.tyust.edu.cn (C.G.); 201921070122@stu.tyust.edu.cn (H.G.); gxx125@126.com (H.W.); zhmliu@tyust.edu.cn (Z.L.); 2011061@tyust.edu.cn (T.Z.)

* Correspondence: lifeizhi@yeah.net; Tel.: +86-13068002395

Table of Content

Characterization of C_nDDGPB (n=8, 10, 12, 14, 16)
Characterization of C_nDDGPB

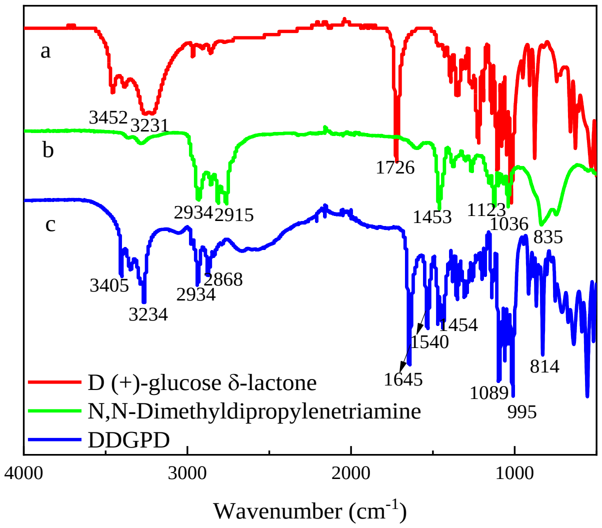

Figure S1. Comparison of infrared spectra of (**a**) D (+) - glucose δ -lactone, (**b**) N,N-dimethyldipropylenetriamine and (**c**) DDGPD comparative IR spectra.

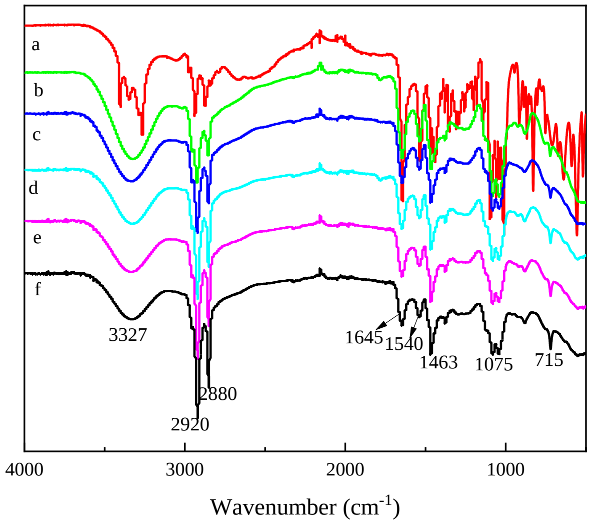

Figure S2. (**a**) DDGPD, (**b**) C_8DDGPB, (**c**) C_{10}DDGPB, (**d**) C_{12}DDGPB, (**e**) C_{14}DDGPB, (**f**) C_{16}DDGPB comparative IR spectra.

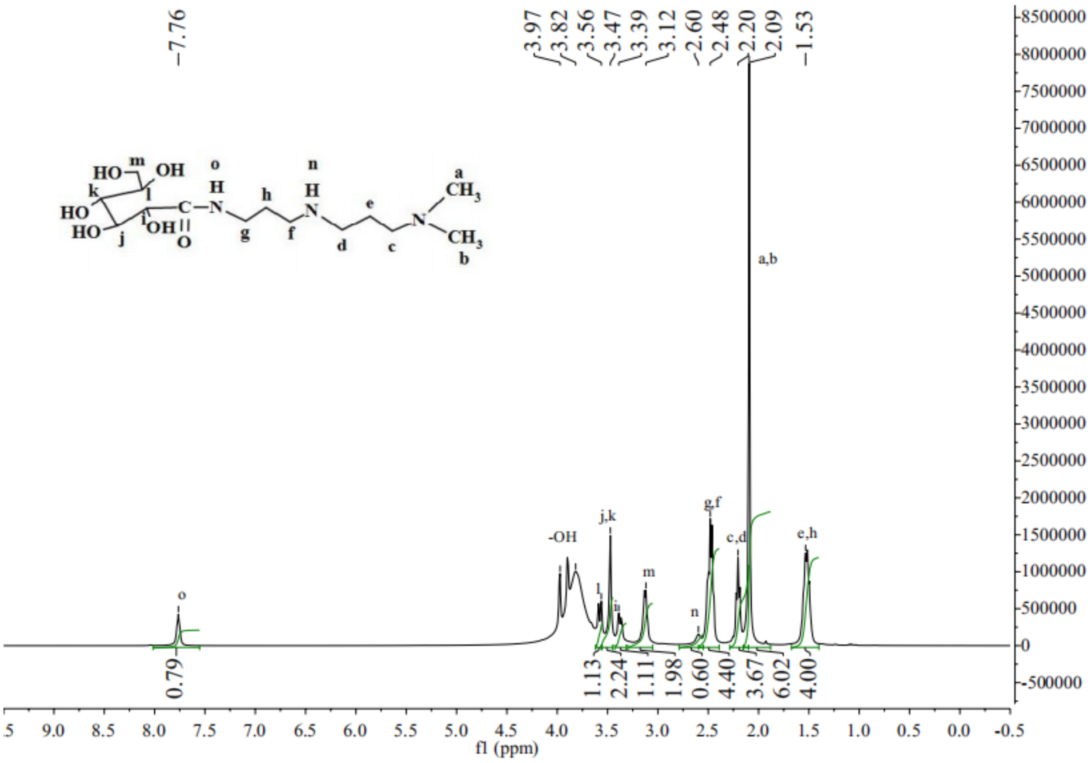

Figure S3. ^{1}H-NMR spectrum of DDGPD.

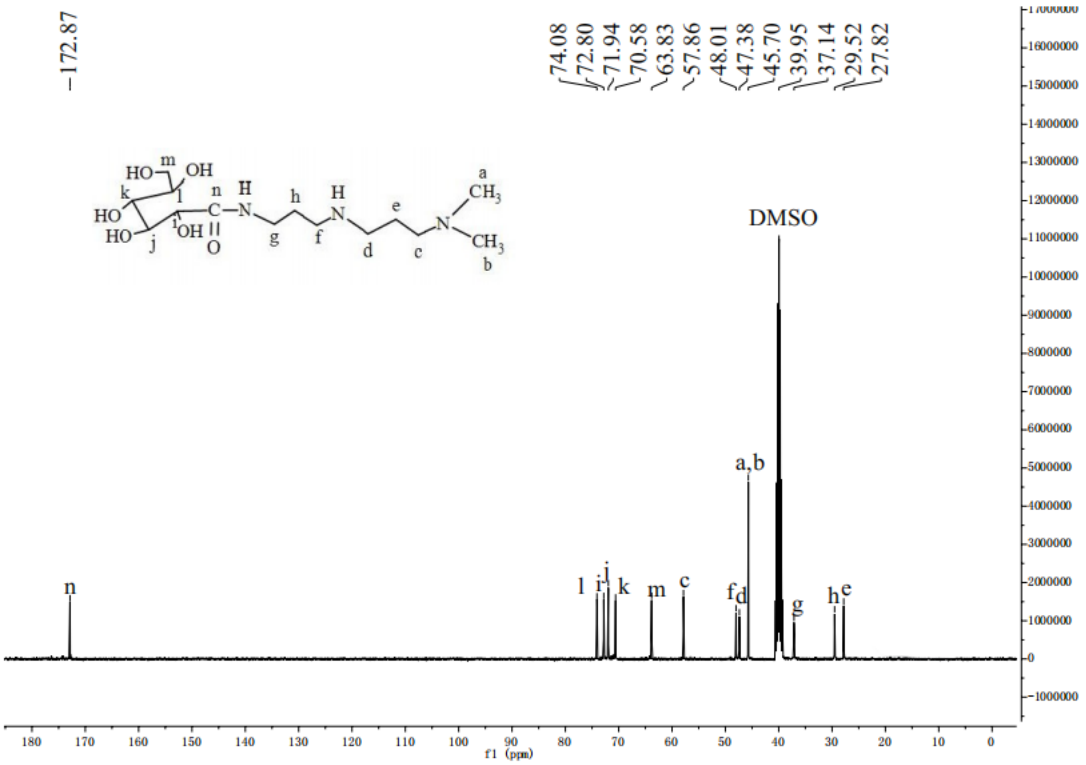

Figure S4. ^{13}C-NMR spectrum of DDGPD.

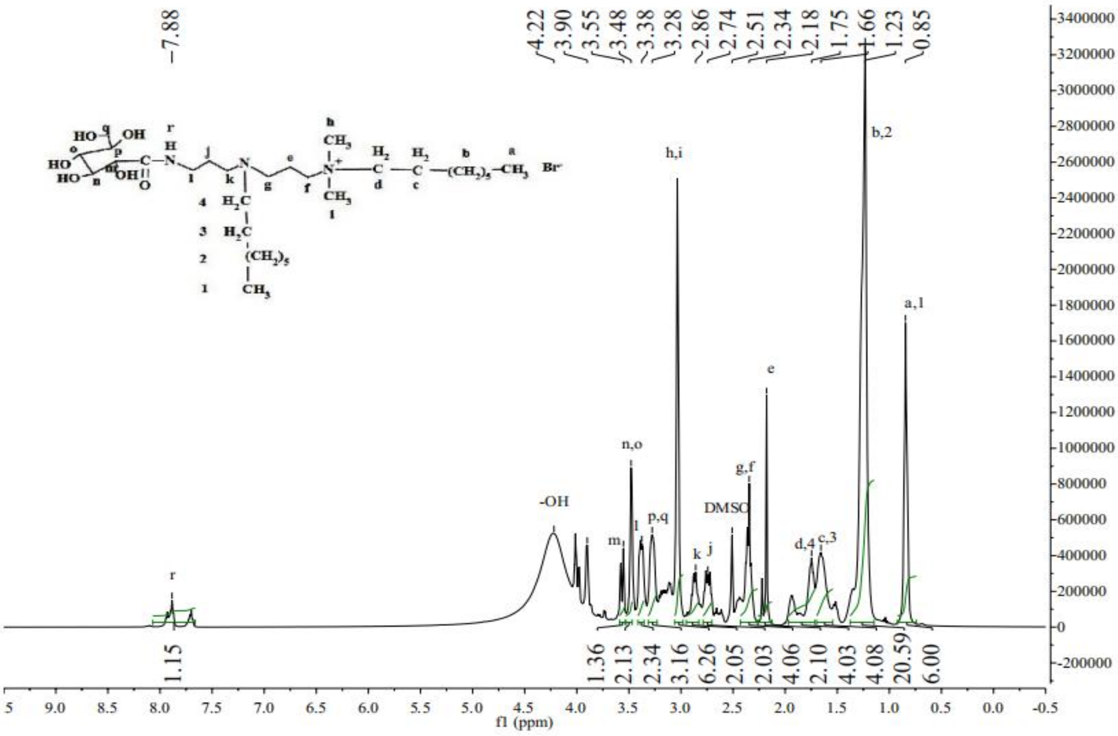

Figure S5. ^{1}H-NMR spectrum of C_8DDGPB.

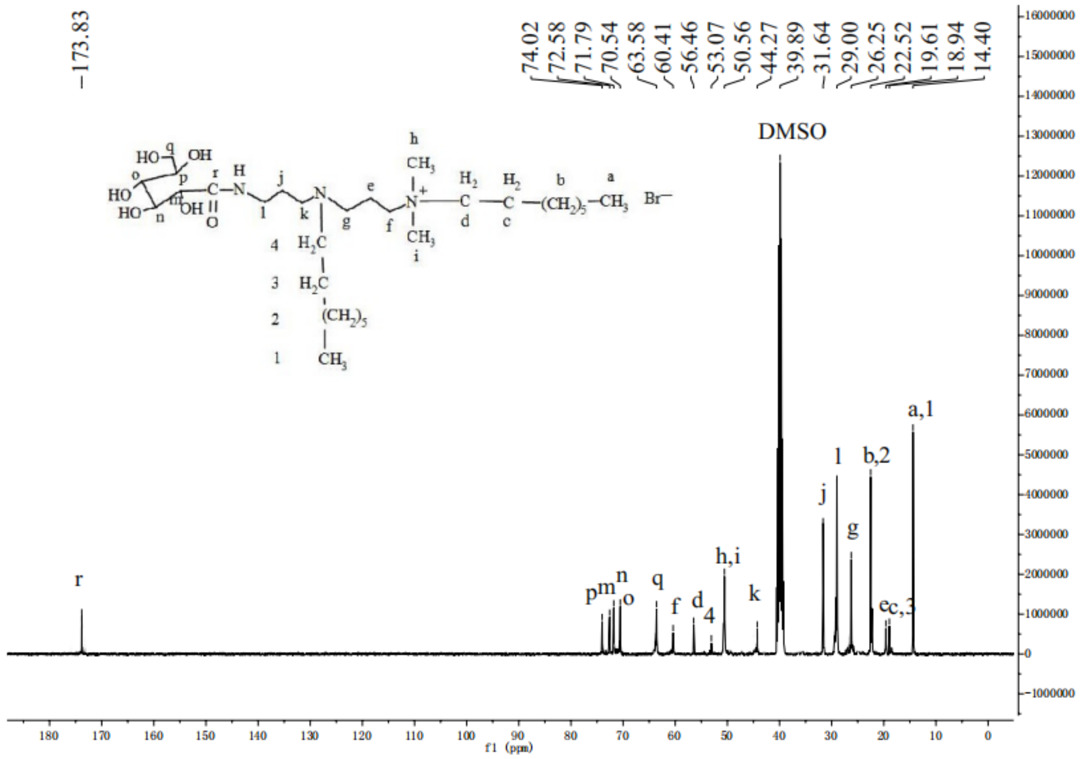

Figure S6. ^{13}C-NMR spectrum of C_8DDGPB.

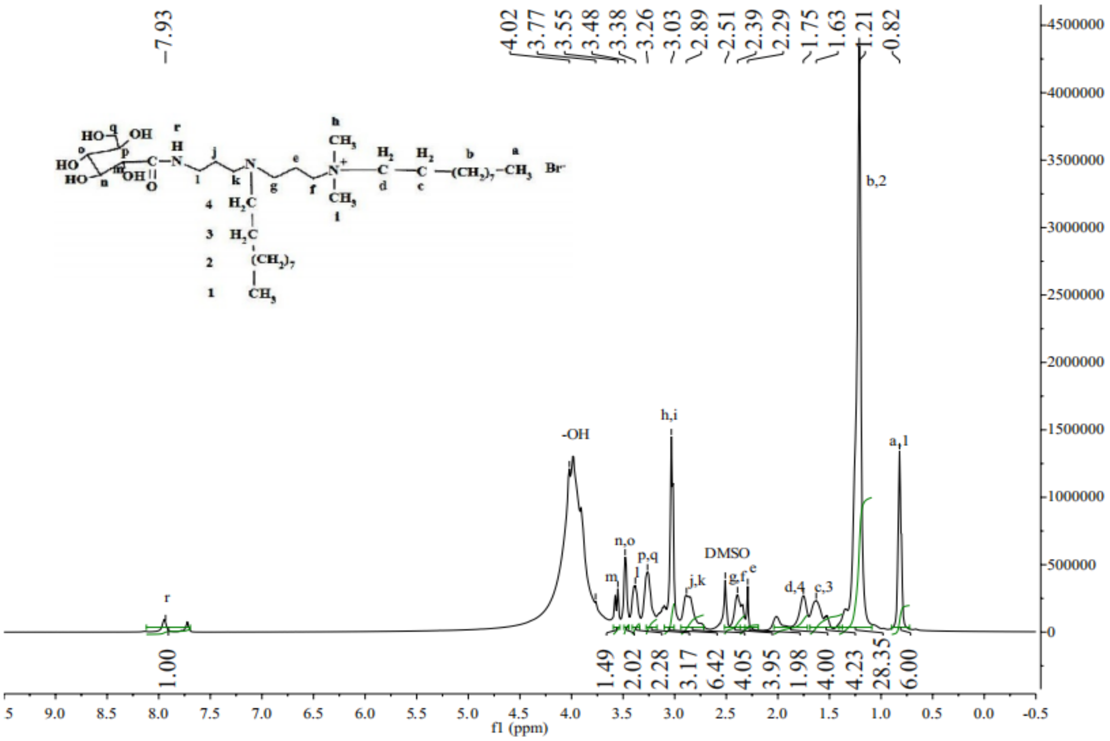

Figure S7. ^{1}H-NMR spectrum of $C_{10}DDGPB$.

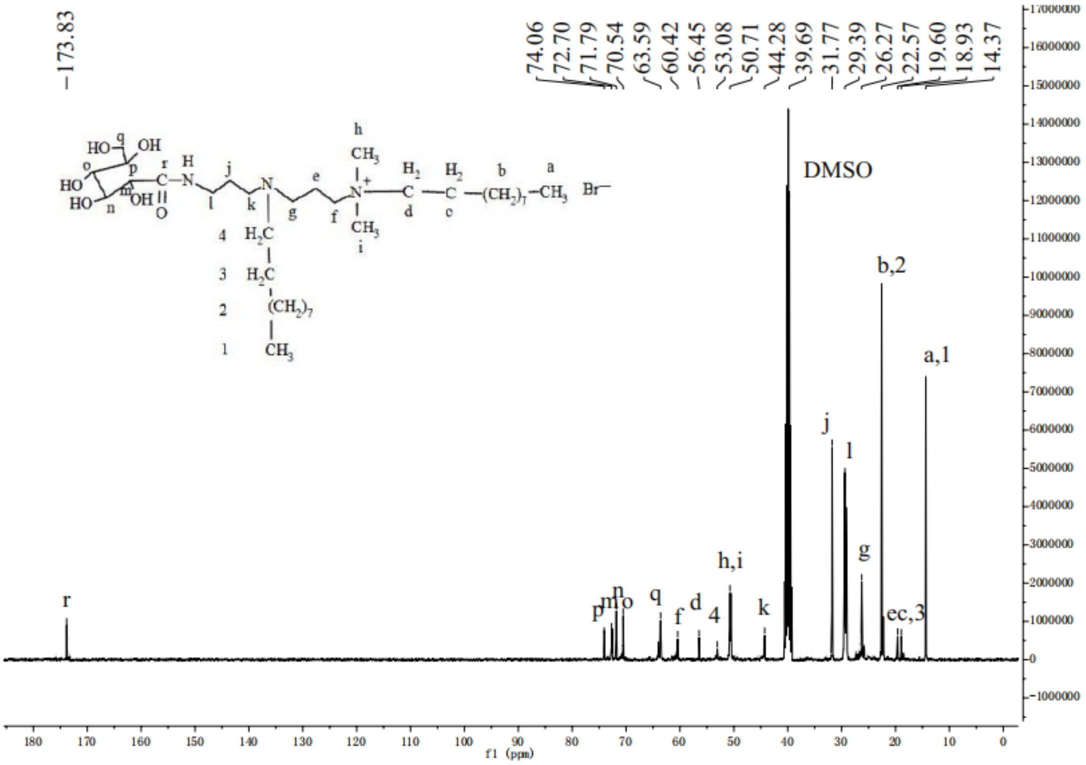

Figure S8. ^{13}C-NMR spectrum of C_{10}DDGPB.

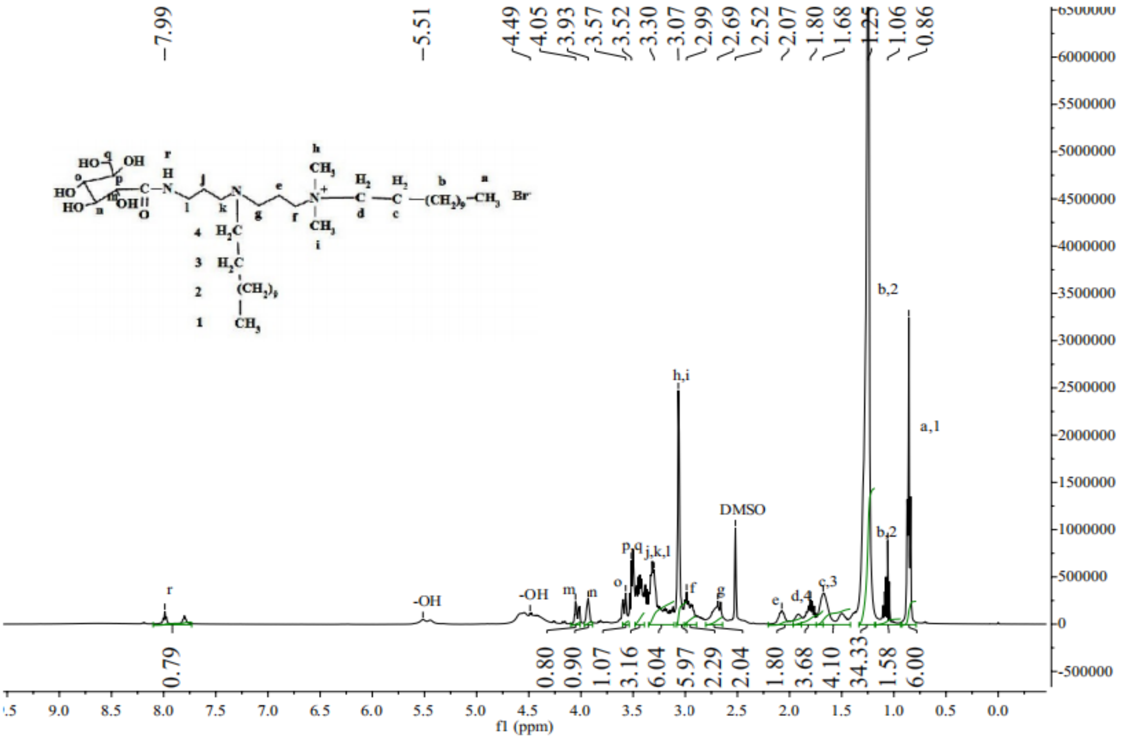

Figure S9. ^{1}H-NMR spectrum of C_{12}DDGPB.

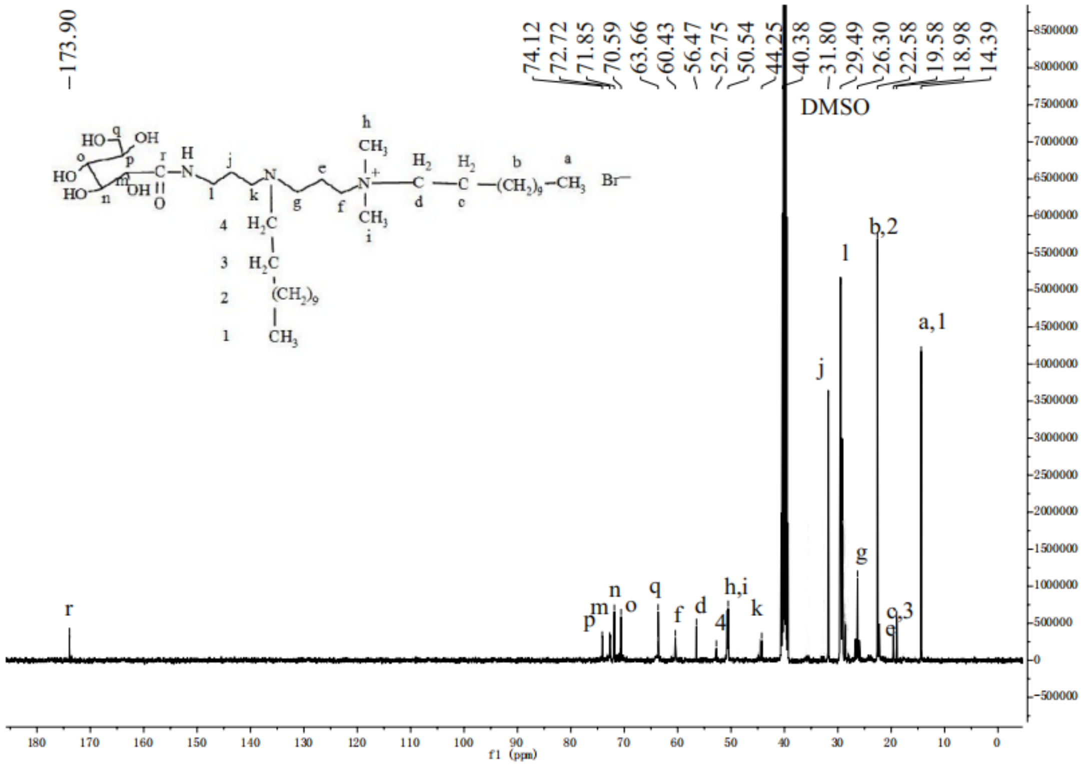

Figure S10. ^{13}C-NMR spectrum of C_{12}DDGPB.

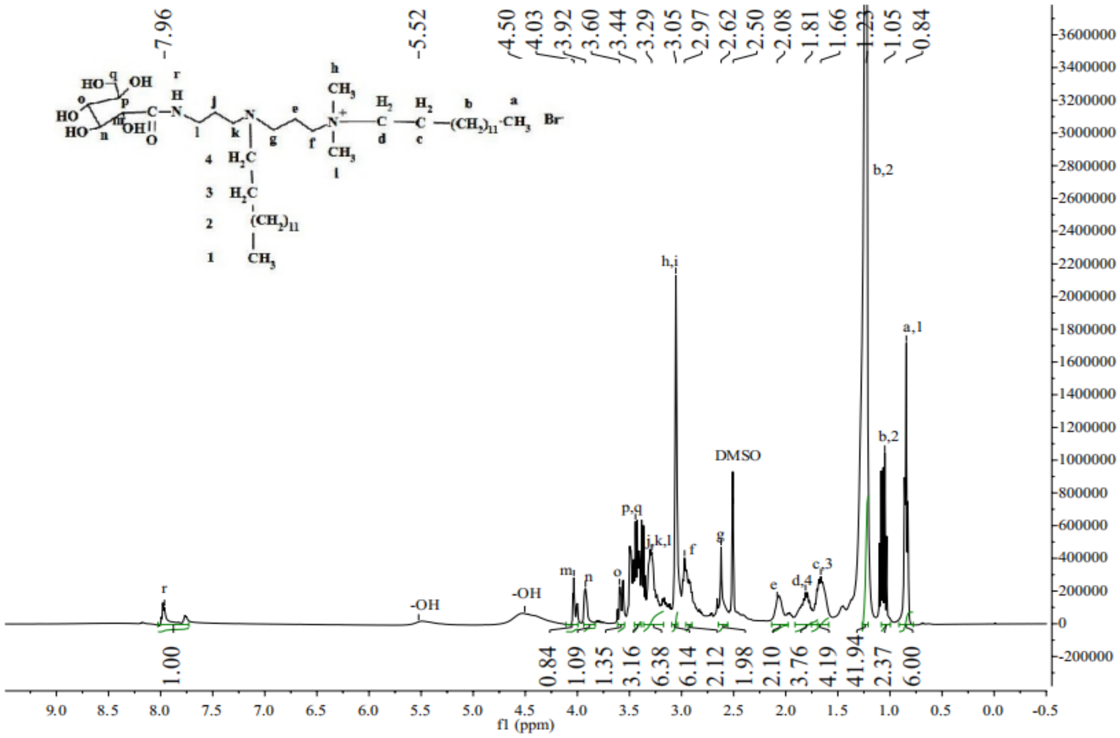

Figure S11. ^{1}H-NMR spectrum of C_{14}DDGPB.

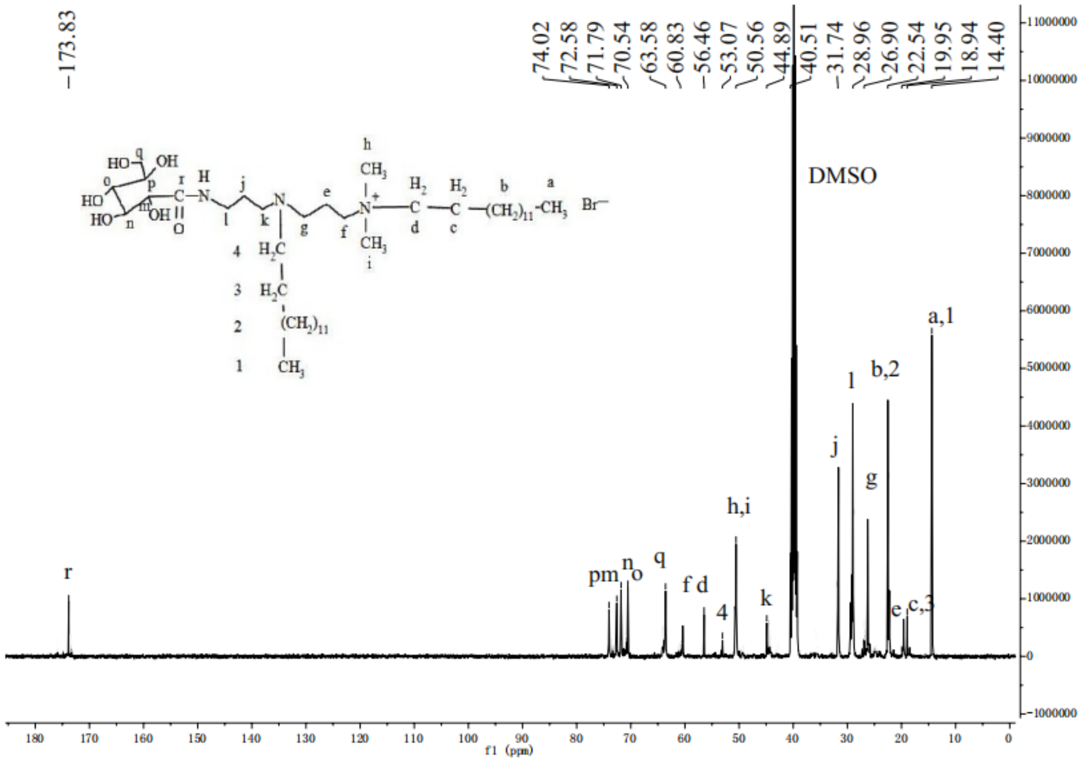

Figure S12. ^{13}C-NMR spectrum of C_{14}DDGPB.

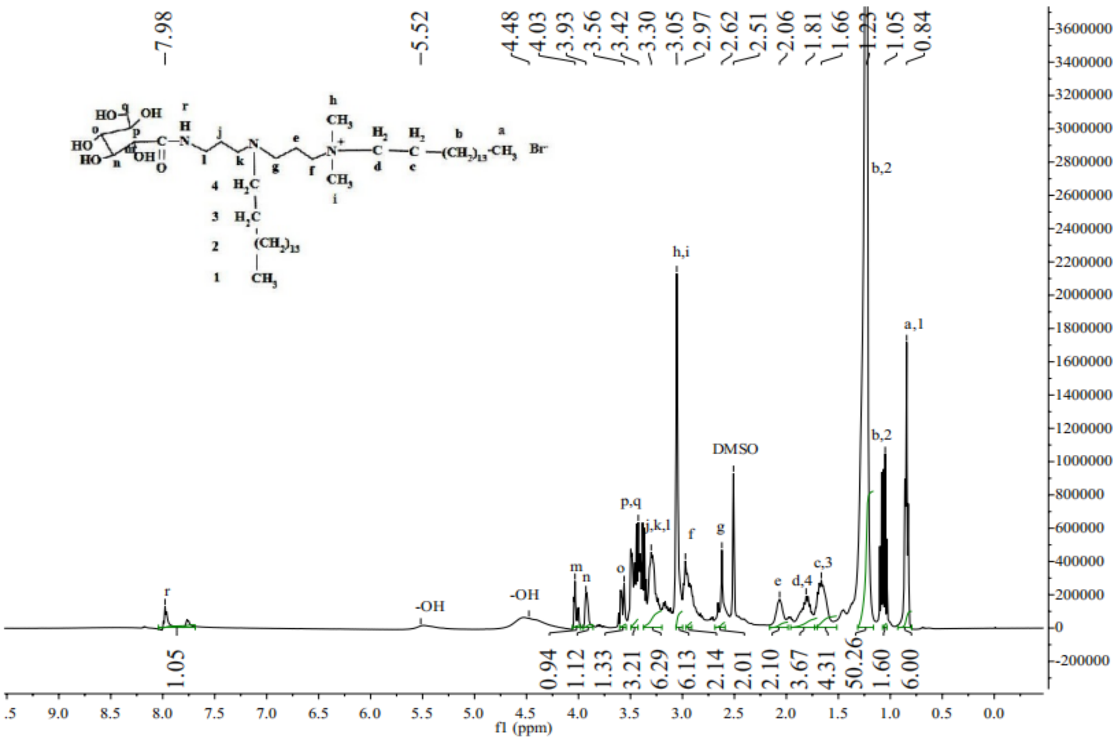

Figure S13. ^{1}H-NMR spectrum of C_{16}DDGPB.

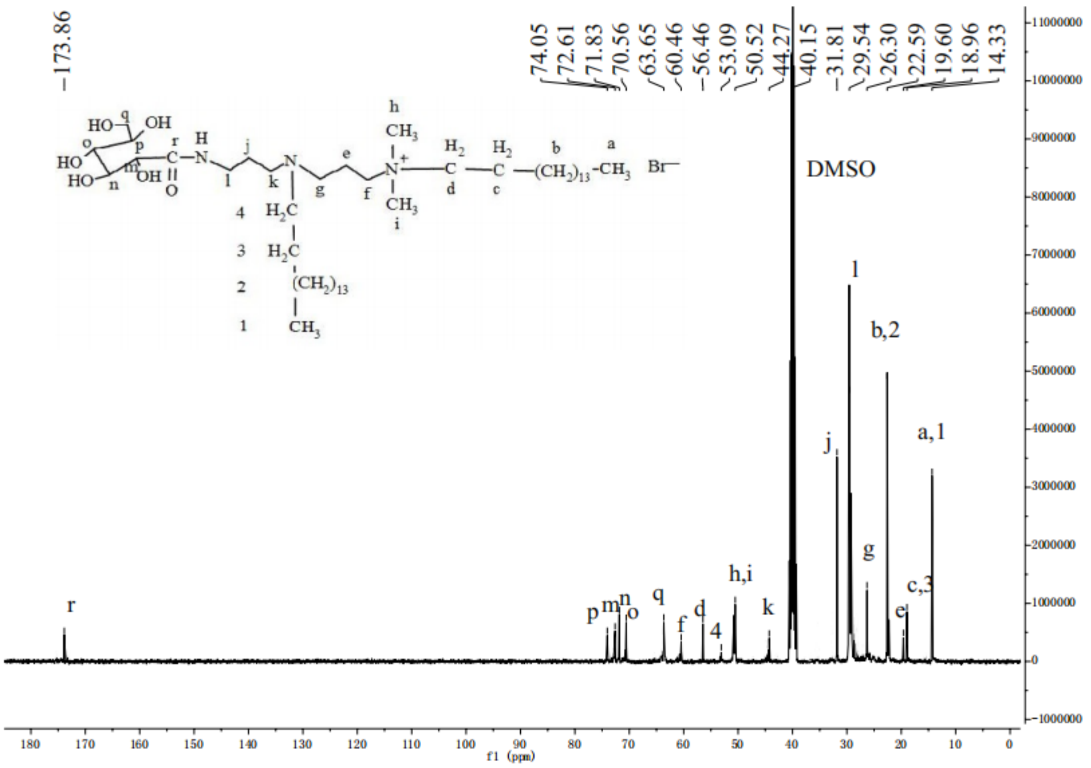

Figure S14. ^{13}C-NMR spectrum of C_{16}DDGPB.
